# Recovery from AKI by KDIGO criteria

**DOI:** 10.1186/cc13576

**Published:** 2014-03-17

**Authors:** D Schrijvers, J Gunst, G Van den Berghe, M Schetz

**Affiliations:** 1 KU Leuven University Hospital, Leuven, Belgium

## Introduction

Data on recovery of AKI are mainly limited to persistent dialysis dependency in patients with dialysis-requiring AKI. The aim of this analysis is to evaluate recovery from different stages of AKI.

## Methods

In a large database (*n *= 4,640) of a previous RCT [1] we estimated renal recovery from AKI defined by KDIGO criteria (without urine output criteria). Patients with end-stage renal disease (*n *= 56), kidney transplantation (*n *= 15) or incomplete data (*n *= 9) were excluded. Patients were classified according to their maximal AKI stage (AKImax) during the ICU stay. Recovery was evaluated by AKI stage at hospital discharge. Complete recovery was defined as the absence of AKI, partial recovery as persistent AKI with a decrease in AKI stage compared with AKImax and no recovery as persistence of AKImax or worsening of AKI after ICU discharge. A persistent 0.3 mg/dl increase of Screat was also considered as no or partial recovery.

## Results

A total of 1,296 patients (28%) developed AKI. AKImax was stage 1 in 580 (45%) (416 with >50% increase of Screat), stage 2 in 207 (16%) and stage 3 in 509 (39%) (348 needing RRT). Mortality increased from 12 to 42% (*P *< 0.0001), hospital stay increased from 21 (14 to 37) to 34 (17 to 63) days (*P *< 0.0001) and complete recovery in survivors decreased from 82 to 53% (*P *< 0.0001) with increasing severity of AKI. In patients requiring RRT, 51% of survivors left the hospital without AKI whereas 16% remained dialysis dependent. Within the AKI 3 group, the need for RRT significantly increased mortality (*P *= 0.0002), but did not affect complete recovery in survivors (51% vs. 58%, *P *= 0.16). Patients with a '0.3 mg/dl increase of serum creatinine only' had a significantly higher mortality than patients without AKI in the |Cu (*P *= 0.0004). They also had a worse kidney outcome at hospital discharge (*P *= 0.006).

## Conclusion

Increasing severity of AKI according to the KDIGO criteria is associated with increased mortality and decreased recovery of kidney function. The need for RRT significantly increases mortality but complete recovery in survivors of AKI 3 is not different with or without RRT. The 0.3 mg/dl criterion proves valid with regard to mortality and kidney outcome.
